# Computed Tomographic Findings in Lemmel Syndrome: A Rare Cause of Obstructive Jaundice

**DOI:** 10.7759/cureus.74533

**Published:** 2024-11-26

**Authors:** Smarth Nathyal, Sugandhi Malgotra, Manik Mahajan, Vikrant Gupta

**Affiliations:** 1 Radiology, Government Medical College, Jammu, Jammu, IND; 2 Radiology, Government Medical College & Hospital, Jammu, Jammu, IND

**Keywords:** biliopathy, computed tomography, diverticulum, ercp, obstructive jaundice

## Abstract

Introduction: Obstructive jaundice resulting from a duodenal diverticulum is known as Lemmel syndrome. Lemmel syndrome should be included in the differential diagnosis in patients presenting with obstructive jaundice in the absence of choledocholithiasis, mass, or a stricture.

Aims and objectives: To describe the computed tomography (CT) findings in patients with Lemmel syndrome.

Materials and methods: Eighteen cases with incidentally detected peri-ampullary duodenal diverticula were retrospectively reviewed over a period of one year. Out of these, eight patients who presented to the hospital with jaundice with or without acute abdominal symptoms were included in the study. CT scans were acquired using the Philips Incisive CT 128 Slice machine, and the findings were recorded.

Results: Jaundice was the presenting complaint in eight patients (100%) followed by fever in four (50%) patients and abdominal pain in three (37.5%) patients. Vomiting was observed in one patient (12.5%). All patients had evidence of obstructive biliopathy on baseline blood investigations. On CT, all patients had periampullary duodenal diverticula arising from the second part of the duodenum along with dilated common bile duct and mild intrahepatic bilobar biliary dilatation. Mild wall thickening and/or enhancement of bile ducts were seen in four patients, while evolving cholangitic abscesses were seen in two patients. Findings of acute pancreatitis and pneumobilia were observed in each patient.

Conclusion: Lemmel syndrome should be suspected in a patient with features of obstructive jaundice when choledocholithiasis, pancreatobiliary tumors, and strictures have been ruled out. Computed tomography remains the preferred imaging modality because of its easy accessibility, less time consumption, and non-invasive nature.

## Introduction

Peri-ampullary duodenal diverticula are mostly asymptomatic, and only 5-10% of affected patients show complications. Duodenal diverticula usually involve the mucosal layer and occur most frequently in the second portion of the duodenum. Depending on the location of the major duodenal papilla relative to the diverticulum, it can be characterized into three separate types: within the diverticulum (type I), within the margin of the diverticulum (type II), or near the diverticulum (type III) [1].

Lemmel syndrome is a term used to describe features of obstructive biliopathy resulting from a peri-ampullary duodenal diverticula, which usually arises along the medial aspect of the second or third part of the duodenum. It was first described by Lemmel in 1934 as a rare cause of obstructive jaundice due to compression of the distal common bile duct by a periampullary duodenal diverticulum [2]. Lemmel syndrome should be kept as a differential diagnosis in patients presenting with obstructive jaundice in the absence of choledocholithiasis or pancreaticobiliary tumors or strictures. Despite its description nearly 100 years ago, only a handful of reported cases of Lemmel syndrome with varying presentations and treatment courses have been described in the literature. In this article, we describe CT findings in eight patients with Lemmel syndrome.

## Materials and methods

Study design

This retrospective observational study was carried out in the postgraduate department of radio diagnosis and imaging in a tertiary care center in North India. The study was approved by the Institutional Ethics Committee of the Institute.

Study population

The study population included all the patients who underwent CECT of the abdomen in the emergency section of our department over a period of one year from June 2022 to June 2023. Out of these, 18 patients with periampullary duodenal diverticula were identified on CECT. From the medical records of the patients, relevant history was obtained, and laboratory investigations were recorded.

Inclusion criteria

Patients with duodenal diverticulum on CECT scans and presenting with acute hepatobiliary symptoms in the emergency department were included.

Exclusion criteria

Patients with motion artifacts and patients with incomplete laboratory workups were excluded from the study.

Methods

CT scans were acquired using the Philips Incisive CT 128 Slice machine. Non-enhanced images and portal venous phase images following intravenous non-ionic iodinated contrast were obtained in all patients from the level of diaphragm to the symphysis pubis. Scans with positive and neutral oral contrast were included in the study. The images were reviewed by three radiologists. Data were presented in the form of tables and expressed as percentages and proportions using MS Excel (Microsoft® Corp., Redmond, WA).

## Results

Eighteen patients with periampullary duodenal diverticula were identified, and amongst these, eight patients with acute hepatobiliary complaints underwent further workup. The age range of the patients was 29-72 years, with a mean age of 51.37 years. The female-to-male ratio of 3:1 (six females and two males). Jaundice was the most common presenting complaint seen in all eight patients (100%). This was followed by fever in four (50%) patients and abdominal pain in three (37.5%) patients. Vomiting and abdominal discomfort were observed in one (12.5%) patient. All the patients underwent baseline blood investigations, which revealed features of obstructive biliopathy (Table 1).

**Table 1:** Clinical features and laboratory investigations in eight cases.

Age/Sex	Presenting complaints	Liver Function Tests						CBC
		S. Bil (mg/dL) (0.2-13)	S. ALP (U/L) (13-126)	SGOT (U/L) (9-40)	SGPT (U/L) (13-40)	S. Amylase (U/L) (19-86)	S. Lipase (U/L) (7-59)	WBC (cells/mm^3^) (4,500-11,000)
35/F	Jaundice, Fever, Epigastric pain	4.3	201	189	175	78	45	15,754
42/F	Jaundice, Epigastric pain, Recurrent vomiting	5.1	219	196	170	457	194	11,050
72/F	Jaundice, Fever	7.9	470	254	214	94	53	16,198
59/M	Jaundice	4.7	195	177	150	60	42	7,400
45/M	Upper abdominal discomfort, Jaundice	5.4	315	172	169	76	57	6,300
62/F	Jaundice	4.9	225	143	160	85	44	5,800
29/F	Jaundice, Fever, Upper abdominal pain	7.9	325	179	185	60	32	17,856
67/F	Jaundice, Fever	9.2	489	197	167	59	34	22,000

**Figure 1:**
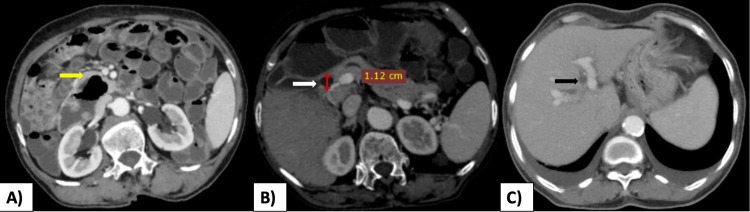
A) Axial CECT image showing a large periampullary duodenal diverticulum (yellow arrow) arising from the second part of the duodenum. B) Dilated proximal CBD (white arrow with red calipers showing measurement). C) Bilobar IHBRD’s (black arrow) are also seen.

All symptomatic patients had elevated serum bilirubin levels, elevated serum alkaline phosphatase levels, elevated serum glutamic-oxaloacetic transaminase levels, and elevated serum glutamic-pyruvic transaminase levels suggestive of an obstructive biliopathy picture. Dilated common bile duct (CBD) and intrahepatic biliary radicles (IHBRs) were seen in all patients on USG. On CECT, periampullary duodenal diverticula arising from the second part of the duodenum were observed in all the patients (Figure 1A). On CECT, dilated CBD and IHBRs were seen in all the patients (Figures 1B, 1C). The largest diverticulum was observed in a symptomatic female patient who presented with a clinical picture of cholangitis.

**Figure 2:**
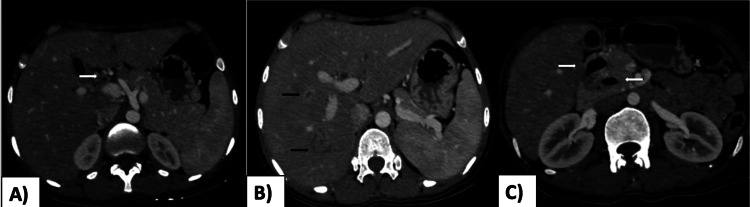
A) Axial CECT images in a 35-year-old female patient presenting with jaundice, fever, and right upper quadrant pain, revealed enhancing thick-walled CBD (white arrow). B) Axial CECT image showing small intrahepatic peripherally enhancing cholangitic abscesses (black arrows). C) Two periampullary duodenal diverticula are also seen (white arrows) with the distal diverticula causing mass effect and resultant CBD dilatation.

Four (50%) patients had elevated white blood cell counts suggesting a diagnosis of cholangitis. USG revealed mild wall thickening of the CBD in two patients. No evidence of any choledocholithiasis/mass was found. On CECT, diffuse circumferential enhancing wall thickening of CBD (Figure 2A) and common hepatic duct was observed in four patients (Table 2). Small cholangitic abscesses were also seen in two patients on CECT, which were missed on USG (Table 2) (Figure 2B).

**Table 2:** Computed tomographic findings in eight cases.

Findings	Number of patients	Percentage (%)
Dilated CBD and IHBRD’s	8	100
Wall thickening and enhancement of CBD/CHD	2	25
intrahepatic biliary radicle	2	25
Pneumobilia	1	12.5
Pancreatitis	1	12.5

**Figure 3:**
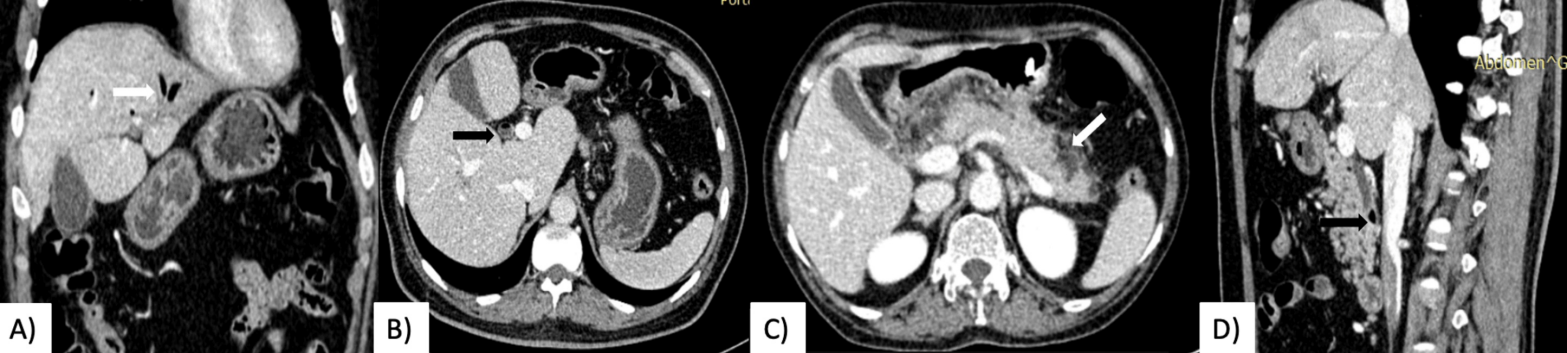
A) Coronal and B) axial CECT images in a patient with duodenal diverticulum showing the presence of air in biliary radicles (white arrow) with associated CBD dilatation (black arrow) s/o pneumobilia. C) Axial and D) sagittal CECT images in a case of acute pancreatitis reveal peripancreatic fat stranding and fluid collections (white arrow) along with a small peri-ampullary duodenal diverticulum (black arrow) with proximal CBD dilatation.

Two patients (25%) had features of obstructive biliopathy only. USG and CECT in these patients revealed dilated CBD with dilated IHBRs. No evidence of any choledocholithiasis/mass was found. One patient had evidence of pneumobilia on CECT (Figures 3A, 3B).

One patient presented with elevated serum amylase and lipase levels in addition to obstructive biliopathy suggestive of acute pancreatitis. USG in this patient revealed features of obstructive biliopathy along with a bulky pancreatic body and tail. No evidence of any choledocholithiasis was found. On CECT, a diagnosis of acute pancreatitis was made (bulky pancreas with peri-pancreatic fat stranding) (Figure 3C) along with a peri-ampullary duodenal diverticula (Figure 3D) and dilated bile ducts.

## Discussion

Duodenal diverticula may be either true diverticula resulting from prolapse of the entire duodenal wall or false diverticula resulting from protrusion of mucosa and submucosa. The majority of duodenal diverticula arise from the second or third part of the duodenum (approximately 75% arising from the second part of the duodenum). Numerous theories have been proposed explaining the occurrence of clinical manifestations by the duodenal diverticulum. These include mechanical obstruction due to direct compression of the ampulla of Vater by the diverticulum, functional obstruction resulting from the sphincter of Oddi dysfunction caused by the diverticulum, or chronic irritation of the diverticula causing diverticulitis, leading to chronic inflammation of the papilla [1,3]. The duodenal diverticulae may be intramural or extramural. The intramural diverticula are congenital and result from incomplete canalization of the duodenal wall [4], while the extramural diverticula are acquired diverticula through the site of wall weakness. In imaging studies, the overall prevalence of duodenal diverticula ranges from 1% to 22%, and almost 95% of them are asymptomatic and diagnosed incidentally [5].

Duodenal diverticula may be associated with complications that may be pancreaticobiliary or non-pancreaticobiliary. The pancreaticobiliary complications associated with duodenal diverticula include obstructive jaundice, cholangitis, acute pancreatitis, or recurrent gallstones/common bile duct stones. Non-pancreaticobiliary complications are uncommon and include diverticulitis, hemorrhage, perforation, or fistula formation [6].

Lemmel syndrome is a term used to describe features of obstructive biliopathy resulting from a peri-ampullary duodenal diverticulum after other causes of obstructive jaundice such as choledocholithiasis or pancreaticobiliary or periampullary tumors or strictures have been ruled out [7]. Clinical symptoms of Lemmel syndrome are non-specific and include jaundice, right hypochondrial pain, and recurrent attacks of abdominal pain [8].

Imaging plays a paramount role in the diagnosis of Lemmel syndrome. Barium study may show contrast-filled outpouchings in the medial wall of the duodenum. Ultrasonography is helpful in the diagnosis of bile duct dilatation, but it is less helpful in the evaluation of diverticula due to the presence of air. Cross-sectional imaging is the mainstay in the diagnosis of Lemmel syndrome. CECT is the imaging modality of choice because of its rapid diagnosis, non-invasive nature, easy accessibility, and multiplanar reconstructions allowing precise evaluation of the diverticulum. The diverticula appear as thin-walled cavitating lesions at the level of the second portion of the duodenum causing compression of the intrapancreatic common bile duct [6,9]. The walls of the diverticulum demonstrate mild homogeneous enhancement, predominantly in the venous phase [8]. The use of oral contrast may also be necessary for evaluating the size of the diverticulum. CECT can also rule out any small peri-ampullary carcinoma or papillitis as the cause of the obstructive jaundice. Magnetic resonance cholangiopancreatography may also help in the diagnosis of duodenal diverticula; however, its use is limited due to the availability and cost of the investigation and is less suited in a developing nation like India.

Endoscopic retrograde cholangiopancreatography (ERCP) offers a diagnostic and therapeutic option in the management of Lemmel syndrome. In asymptomatic high-risk patients, sphincterotomy and stent placement with ERCP are recommended. Because of its invasive nature, it still remains reserved only as a therapeutic option rather than a diagnostic option [7]. In patients whose Lemmel syndrome is caused by chronic papillary fibrosis or sphincter of oddi dysfunction, biliary obstruction is relieved by endoscopic sphincterotomy.

Imaging differentials of Lemmel syndrome include type II or type III choledochal cysts, pancreatic pseudocysts or infected peri-pancreatic collections, peri-ampullary neoplasms, and necrotic lymph nodes. Detailed history and CECT are sufficient to exclude the differential diagnosis.

This study had a few limitations. Firstly, it was a retrospective observational study only. Secondly, the sample size was small as only eight symptomatic patients with duodenal diverticulum were included in the study. Thirdly, MRCP and ERCP follow-ups were unavailable.

## Conclusions

Lemmel syndrome should be considered a differential diagnosis in a patient with features of obstructive jaundice when choledocholithiasis, pancreatobiliary tumors, and strictures have been ruled out. USG can often miss the diagnosis of duodenal diverticula. CECT is the most sensitive imaging modality in suspected cases of Lemmel syndrome. Timely diagnosis of Lemmel syndrome is crucial to achieve accurate patient care management and to avoid complications of delayed management.
